# Developing Patient-Centered Inflammatory Bowel Disease–Related Educational Videos Optimized for Social Media: Qualitative Research Study

**DOI:** 10.2196/21639

**Published:** 2020-10-20

**Authors:** Carine Khalil, Welmoed Van Deen, Taylor Dupuy, Nirupama Bonthala, Christopher Almario, Brennan Spiegel

**Affiliations:** 1 Division of Health Services Research Center for Outcomes Research and Education Cedars-Sinai Medical Center Los Angeles, CA United States; 2 LIRAES Paris Descartes University Paris France; 3 Department of Medicine Cedars-Sinai Medical Center Los Angeles, CA United States; 4 Division of Gastroenterology and Hepatology Cedars-Sinai Medical Center Los Angeles, CA United States

**Keywords:** inflammatory bowel disease, educational videos, patient education, design thinking, qualitative research, mobile phone

## Abstract

**Background:**

Important knowledge gaps have been identified related to the causes and symptoms of inflammatory bowel disease (IBD) and medical treatments and their side effects. Patients with IBD turn to social media to learn more about their disease. However, such information found on the web is misleading and often of low quality.

**Objective:**

This study aims to gain an in-depth understanding of the unmet educational needs of patients with IBD and to use the resulting insights to develop a collection of freely available, evidence-based educational videos optimized for dissemination through social media.

**Methods:**

We used design thinking, a human-centered approach, to guide our qualitative research methodology. We performed focus groups and interviews with a diverse sample of 29 patients with IBD. Data collection was performed in 3 phases (inspiration, ideation, and implementation) based on IDEO design thinking. Phase 1 offered insights into the needs of patients with IBD, whereas phases 2 and 3 involved ideation, prototyping, and video testing. A thematic analysis was performed to analyze the resulting data.

**Results:**

Patients emphasized the need for educational videos that address their challenges, needs, and expectations. From the data analysis, 5 video topics and their content emerged: IBD treatments’ risks and benefits; how to be a self-advocate; how to stay healthy with IBD; how to cope with IBD; and educating families, friends, and colleagues about experiences of patients with IBD.

**Conclusions:**

Design thinking offers a deep understanding and recognition of the unmet educational needs of patients with IBD; this approach informed the development of 5 evidence-based educational videos. Future research will formally test and disseminate these freely available videos through social media.

## Introduction

### Background

Inflammatory bowel diseases (IBD), including Crohn disease and ulcerative colitis, are inflammatory conditions of the intestines that can cause debilitating symptoms and decrease patients’ quality of life [1,2]. Accurate information and education are important aspects of IBD treatment, as they can improve the quality of care and help patients cope with IBD-related worries and concerns [3,4]. However, critical knowledge gaps have been identified among patients with IBD, including lack of knowledge about the disease’s causes and symptoms and medical treatments and side effects [5-7]. Consistently, a large Swiss study assessing informational needs and concerns in 728 patients with IBD highlighted that the information patients received about their disease was insufficient [8].

Many patients are unhappy with the information they receive after diagnosis. Although most would prefer to receive educational content through their doctor’s office, their education needs are often not met in this setting [7]. Information provided is commonly based on clinicians’ assumptions of what patients need to know and is not always aligned with patients’ actual needs [4]. Therefore, many patients turn to other sources, including the internet and social media, to obtain additional information, share their experiences, and connect with other people with IBD [4,6,9]. In fact, in our previous study that used social media data to examine patients’ understanding of the risks and benefits of biologics in IBD, more than 25% of posts were from people seeking information and support through the online IBD community [6]. Although there is growing interest among patients to use social media for IBD-related information, information on the web is misleading and often of low quality [9].

Involving patients throughout educational materials development is critical for a deeper understanding of their expectations and addressing their needs. Design thinking—an iterative human-centered approach that emphasizes empathy, collaborative thinking, prototyping, and learning from failure—is well suited for designing interventions from the perspective of those impacted by it [10,11]. This methodology emphasizes the use of qualitative research methods within a structured framework for design purposes and is used across industries to improve product development, user experience, and customer service. Given the increasing focus on patient-centered care, the use of design thinking in health care is also gaining interest. By prioritizing end users’ core needs and continuously integrating their feedback, design thinking offers a way to develop interventions, including digital solutions that are successful, acceptable, and useful to the patients [12-14].

Design thinking has been applied to develop patient- and provider-facing interventions across diverse health conditions, including diabetes, chronic obstructive pulmonary disease, and posttraumatic stress disorder, among others [15]. Although several educational interventions have been developed for IBD, few were informed by patients’ input. In these studies, design thinking was implemented with various degrees of rigor. Not all studies used direct end-user input to assess users’ needs, and none reported brainstorming or ideation sessions, which are essential for the collaborative generation of solution concepts for the target population [4]. In addition, previous studies did not obtain end-user feedback on low-fidelity prototypes [15], an essential step that allows the design-thinking team to get user feedback at an early stage in the intervention-development process [10].

### Objectives

Here, we aim to gain an in-depth understanding of the unmet needs and expectations of patients with IBD for web-based educational videos. We then used these insights to inform the development of a series of short educational videos optimized for dissemination through social media. We applied design-thinking methodology to understand patients’ experiences, identify their challenges, and obtain iterative input on video prototypes during the development process.

## Methods

### Design-Thinking Approach

To guide our qualitative research methodology, we applied a design-thinking model developed by IDEO, a global design-thinking company that creates human-centered products, services, and organizations. This model is widely used to explore solutions for social problems faced by communities and uses 3 iterative phases to design innovative solutions [16]. In the first phase, called the inspiration phase, researchers learn about people’s lives, preferences, expectations, needs, thoughts, emotions, and challenges. It consists of collecting stories and gathering inspiration from patients and requires building empathy to understand what they need and how they behave, feel, and think. In the second phase, called the ideation phase, ideas are generated by the research team, which are then converted to low-fidelity prototypes that can be tested by end users. The third and last phase—the implementation phase—consists of testing high-fidelity prototypes before producing the final product and launching it into the market. Multimedia Appendix 1 shows our study design based on IDEO’s design-thinking model.

#### Phase I–Inspiration Phase

In the inspiration phase, we performed 2 in-person focus groups with 11 patients with IBD and 6 semistructured phone interviews with individual patients with IBD to obtain an in-depth understanding of their preferences, expectations, and unmet educational needs. The focus groups allowed interpersonal discussions to elucidate similarities and differences among participants’ experiences and beliefs. The individual interviews enabled us to conduct in-depth discussions with patients who have different needs and expectations, such as those with higher disease severity or lower literacy levels compared with *typical* patients with IBD.

#### Phase II–Ideation Phase

In the ideation phase, low-fidelity prototypes were developed based on the data synthesized and analyzed in the inspiration phase (phase I). Prototypes consisted of 5 video scripts and character and location prototypes for the videos. The scripts were developed iteratively in a series of ideation sessions with health services researchers, physicians with expertise in IBD, video producers, and patients with IBD. In the first session, the inspiration phase results were presented to all coauthors by a qualitative researcher (CK) on the team. The team then deliberated about potential topics for each of the videos and created outlines of each video's content. The outlines were shared with the video production team, who then developed the initial scripts and prototypes of the videos’ characters and locations. To ensure that the videos were suitable for dissemination through social media, the scripts were designed to be short and concise with an anticipated video length of ≤90 seconds. The initial scripts were first reviewed and revised based on input from the research team. Afterward, 2 in-person focus groups were conducted with 12 patients with IBD, including some of those who participated in the inspiration phase, to gather their feedback on the scripts and character and location prototypes. In an additional ideation meeting, we reviewed participants’ feedback with our multidisciplinary team and incorporated their feedback in designing 5 high-fidelity video prototypes.

#### Phase III–Implementation Phase

In the final implementation phase, high-fidelity prototypes of the videos were developed by the production team based on the final script, which was tested by 10 patients with IBD via in-person interviews. These interviews aimed to test the videos’ practicability and obtain additional insights and feedback from a sample of additional users. Hence, 10 patients with IBD tested the high-fidelity prototypes and made minor comments on their format and content, based on which additional changes were made to the videos. This phase helped us understand how well the 5 videos met patients’ expectations and needs.

### Population

For the inspiration phase (phase I), we included patients with different disease severity, literacy, and digital levels. Both the 2 focus groups included a diverse representation of 5 to 6 *typical* patients with IBD, including participants from diverse age groups, genders, races and ethnicities, and IBD type (Multimedia Appendix 2). By recruiting a diverse sample, we aimed to obtain broad perspectives and insights regarding the needs, expectations, and traits of potential viewers of future videos. For the interviews, we recruited 6 patients with needs and behaviors that differed from those of *typical* patients. We included patients with a particularly mild and severe disease course, a patient with a recent IBD diagnosis, patients with low health and digital literacy, and a patient with reduced access to care. Their inclusion in the study population helped us generate additional insights and identify high-priority issues that need to be addressed in the educational videos (Multimedia Appendix 2).

In the ideation phase (phase II), we recruited a diverse group of patients with IBD for the 2 focus groups (6 patients in each) to obtain feedback on the scripts. This phase included participants who previously participated in the inspiration phase to confirm that we correctly interpreted what they told us and appropriately translated the findings into the video scripts. We also included new patients during this step to ensure the transferability of the results to other patients. Finally, in the implementation phase (phase III), we recruited a convenience sample of 10 patients with IBD who had not previously participated in the study to obtain feedback on the prototyped videos. By recruiting a group of new patients in the final phase, we aimed to collect unbiased perspectives.

### Data Collection

Semistructured interview guides were developed for the focus groups and interviews (Multimedia Appendix 3). All discussions were audiotaped and transcribed with the consent of the participants. For the focus groups, a researcher moderated the sessions while 2 other researchers recorded detailed notes; 2 researchers conducted the interviews. Data on demographics, disease characteristics, medical literacy, and digital literacy were collected using a short survey before the focus groups and interviews.

In the inspiration phase (phase I), we conducted 2 in-person focus groups and 6 phone interviews with patients with IBD. The semistructured interview guide (Multimedia Appendix 3) included open-ended questions such as “What type of challenges did you deal with when you were first diagnosed with IBD?”, “Do you discuss treatment options with your doctor?”, and “How can educational videos help you participate in your treatment decision?”.

In the ideation phase (phase II), 2 in-person focus groups were performed with patients with IBD to gather their opinions and perceptions of the initial video scripts and prototypes of the characters. The semistructured interview guide (Multimedia Appendix 3) included questions such as “What are your thoughts on the content of each video?”, “How does the video improve your knowledge?”, “Does it reduce your anxiety or fear or depression?”, and “What do you think about the animation characters?”.

In the implementation phase (phase III), 10 in-person interviews were conducted to assess their opinions and perceptions of the prototype videos. The interview guide (Multimedia Appendix 3) included both closed- and open-ended questions related to the content and format of each video: “Was the language easy to understand?”, *“*What do you think about the music?”, “How can we improve the videos?”, and “What did you think of the video?”

### Data Analysis

A thematic analysis approach was used to examine the interview and focus group data. The analyses were performed by an experienced researcher (CK) with formal training in qualitative methods. The qualitative data were carefully reviewed and rereviewed to immerse ourselves in the language and obtain a global sense of what patients expressed during the discussions. Throughout the reading, sentences and/or paragraphs were coded, and important sections of texts were highlighted and labeled. Hence, key labels were inductively identified in the unstructured data [17]. After sorting and combining the identified labels, a set of inductive themes and subthemes were defined and justified with verbatim quotes [18]. Table 1 illustrates examples from the coding process in each phase.

**Table 1:** Extracts from the coding process in phases 1, 2, and 3.

Phases	Quotes	Themes
Phase 1–Inspiration	“When you research it’s like one site will say something and the other site will contradict what someone has said…. I’m really trying to change my diet and how to do that is what I’m really searching out right now.”	Conflicting information on the web Looking for diet information
Phase 2–Ideation	“Each drug has so many different risks, but maybe mention it a couple more times in the video so the person knows to get some more information on what the potential risks could be.”	Important to stress the risks associated with each drug
Phase 3–Implementation	“It would be great to show this video to people who have just been diagnosed and are going to the doctor for the first time.” “I thought the video had a good message that you should support people with IBD.”	Perceived usefulness of the video when first diagnosed Satisfaction with the main message of the video

### Ethical Considerations

This study was approved by the institutional review board of the Cedars-Sinai Medical Center under the protocol number Pro55548. Patients were initially approached by their treating physician, who explained the study’s purpose and asked for their permission to be approached by a member of the research team. Information sheets were provided, and verbal consent was obtained from all participants before the focus groups and interviews. Participants were reminded of their right to pass on answering any question or discontinuing the interviews or focus groups at any moment. Participants received a US $100 Amazon gift card at the beginning of the focus groups or interviews.

## Results

### Phase 1–Inspiration Phase

A total of 17 patients with IBD were included in the focus groups (n=11) and one-on-one interviews (n=6) during the inspiration phase. The demographics and clinical characteristics of the patients are shown in Table 2. Although most patients in the focus groups were highly educated and with high digital literacy levels, we purposefully sought to increase diversity in these aspects in the individual interviews (Table 2). The focus groups lasted 2 hours, and interviews lasted between 15 mins and 1 hour. Several key themes were identified, and thematic saturation was achieved after 2 focus groups and 3 interviews.

**Table 2:** Demographics of participants.

Demographic		Phase 1–Inspiration		Phase 2–Ideation	Phase 3–Implementation
		Focus groups (n=11)	Interviews (n=6)	Focus groups (n=12^a^)	Interviews (n=10)
Female gender, n (%)		8 (73)	5 (83)	8 (67)	8 (80)
Age, (years), median (range)		41 (22-83)	34 (21-64)	41 (23-83)	25 (36-52)
**Race, n (%)**					
	Black	4 (36)	2 (33)	4 (33)	0 (0)
	Native Hawaiian or other Pacific Islander	0 (0)	0 (0)	1 (8)	0 (0)
	White	4 (36)	2 (33)	6 (50)	10 (100)
	Multiracial	2 (18)	0 (0)	1 (8)	0 (0)
	Other	1 (9)	2 (33)	0 (0)	0 (0)
	Hispanic ethnicity	1 (9)	2 (33)	2 (17)	0 (0)
**Insurance^b^, n (%)**					
	Employer-sponsored	9 (82)	3 (50)	9 (75)	8 (80)
	Marketplace	1 (9)	1 (17)	1 (8)	0 (0)
	Medicaid	0 (0)	2 (33)	1 (8)	2 (20)
	Medicare	2 (18)	0 (0)	2 (17)	0 (0)
**Highest education, n (%)**					
	High school	0 (0)	3 (50)	0 (0)	3 (30)
	College degree	4 (36)	3 (50)	6 (50)	4 (40)
	Graduate degree	7 (64)	0 (0)	9 (50)	3 (30)
**Computer or smartphone use, n (%)**					
	Every day	11 (100)	5 (83)	11 (92)	10 (100)
	A few times per week or month	0 (0)	0 (0)	0 (0)	0 (0)
	Occasionally	0 (0)	0 (0)	1 (8)	0 (0)
	Never	0 (0)	1 (17)	0 (0)	0 (0)
**Type of IBD^c^, n (%)**					
	Crohn disease	8 (36)	4 (67)	9 (75)	7 (70)
	Ulcerative colitis	3 (27)	2 (33)	3 (25)	3 (30)
Disease duration (years), median (range)		15 (2-39)	12.5 (1-22)	17.5 (2-39)	19 (2-36)

^a^10 participated in phase 1 and phase 2.

^b^Multiple answers may apply.

^c^IBD: inflammatory bowel disease.

#### Patients’ Web Experience

Participants emphasized the need for educational videos that address patients’ challenges, needs, and expectations: “I think it’s actually really great and helpful [to develop educational videos for IBD], and I think it would be helpful to tackle it in multiple ways…” Moreover, developing videos tailored for social media was aligned with patients’ behavior and their extensive use of the internet: “before, they would send you home with a brochure… but now it’s the Internet… [I am] on Facebook all the time, every day…” However, participants reported conflicting experiences with the information they found on the web. On the one hand, IBD-related information on the web was perceived as overwhelming and unreliable: “there’s so much stuff on the Internet... some of it is just conflicting.” This affected their trust in information on the web (“I don’t trust the Internet, there’s a lot going on”; “you can also get conflicting information”) and created “panic and anxiety” for some people with IBD. In this regard, our participants underlined the importance of using reputable sources such as Mayo Clinic, Harvard Medical School, and WebMD to get the information they needed to manage their disease. On the other hand, participants noted that the internet was helpful and supportive: “it’s really nice that there is support at our fingertips with a bunch of people with shared experiences.” It helped patients “feel better and less alone” as it made them realize “how many other people out there are dealing with this [IBD].” Patients used the internet to seek information about nutrition, healthy recipes, alternative remedies, and lifestyle choices. They also used the internet to find articles related to patients’ shared experiences, advances in the field of IBD, and available medication options and their associated side effects.

#### Concerns Regarding IBD Treatments

Concerns were expressed by participants regarding the use of biologics and its side effects:

I remember being really nervous about starting biologics

the side effect of medication is worse than the disease

Patients also noted a need to learn about the different treatment options and their associated risks and benefits:

It’s good to know what the risks are so that you are informed

It’s all about the pros and cons.

Learning about medication risks and benefits helped patients make informed treatment decisions:

now that you have the information, you can take it and you can make decisions.

#### Perceived Importance of Self-Advocacy

Patients also emphasized the importance of self-advocacy in managing their IBD. They often struggled to get IBD medications approved by their insurance and delivered on time:

It’s a hassle to get the medication

I missed a few months because the insurance wasn’t happening

They also described their experiences with insurance companies as being *tough*, *a hassle*, *a nightmare*, *a hell of a process*, *worrying*, and *stressful*;

it’s really tough to work with the insurance

I think insurance is a nightmare and it's so stressful;

I was getting a little worried like I don't know if my insurance will cover it.

Therefore, participants expressed the need to be persistent and self-advocate to get the right medication and dosage approved:

You have to be persistent with them

insurance

I think that providing people with insights and tips on how to be aggressive about it [getting the injections] would be very helpful.

They also reported that it is essential for patients with IBD to feel comfortable sharing their symptoms, concerns, and questions with their providers:

you could have stopped it from happening if you talked to your doctor;

letting them [doctors] know what you found out.

#### Need for a Healthy Lifestyle

Patients with IBD often looked for health information in brochures from their doctor’s office or on the web:

I am on Facebook daily, so every now and then I see an article about IBD that catches my eyes, I’ll click on it and read it.

They believed that their IBD symptoms were highly affected by their lifestyle choices. Hence, it was important for them to learn how to adjust their lives and keep their bodies in shape. They expressed the need to develop a video that highlights the benefits of exercising and includes recommendations for a healthy diet and lifestyle:

I think that it would be helpful to tackle it in multiple ways such as telling people types of foods to eat, recipes, lifestyle choices.

They also reported that the video should emphasize the importance of physical activities and meditation to alleviate stress.

#### Importance of Mental Support

Participants also pointed out that they can feel overwhelmed and alone with their illness and that they believe that IBD is also a mental disease. Hence, they wanted tips on coping with their IBD diagnosis and living with their condition: “no matter how much medicine you take, if you don’t have a positive mental state it won’t help.” Patients often sought support from people who also have IBD or other autoimmune diseases:

I just went online finding people with IBD;

I keep this close-knit circle of people with autoimmune disease.

It was also important to participants that the video highlighted that there are many effective treatments for IBD, patients with IBD have a normal life expectancy, and other people are also going through it: “when I see another person is experiencing the same thing I feel less alone.”

#### Perceived Lack of Understanding From Their Surroundings

Furthermore, our participants highlighted the lack of understanding they perceived from their families, friends, and colleagues:

they really don’t understand; they think you are just not being sociable;

because we look normal that’s the problem;

what you see on the outside is not what’s happening on the inside.

Therefore, they expressed the need to improve others’ understanding of what patients with IBD go through: “you can’t eat like everyone else, they don’t get it.”

### Phase 2–Ideation Phase

**Figure 1:**
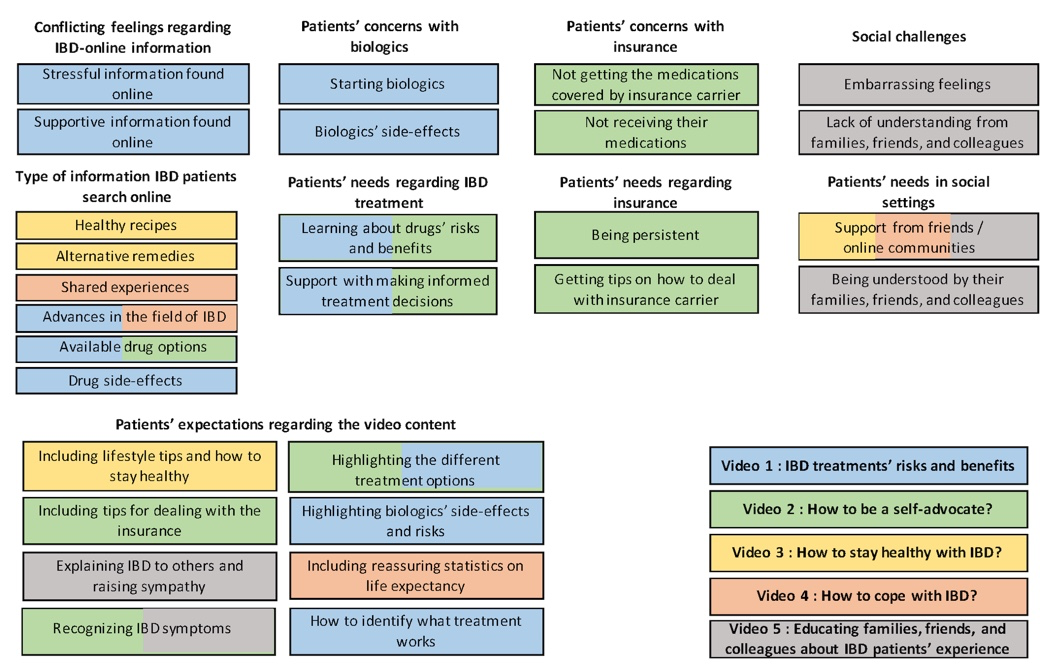
The emergence of 5 video topics based on the themes identified in phase 1. IBD: inflammatory bowel disease.

In the ideation phase, we interpreted the data obtained in the inspiration phase to blueprint the content of the 5 educational videos. The following 5 topics emerged: (1) IBD treatments’ risks and benefits, (2) how to be a self-advocate, (3) how to stay healthy with IBD, (4) how to cope with IBD, and (5) educating families, friends, and colleagues about experiences of patients with IBD. Figure 1 shows how the themes and subthemes identified in phase 1 mapped to each video topic.

On the basis of these findings, 5 video scripts and character prototypes were developed, which were reviewed by 12 patients with IBD during 2 focus groups (Table 2). Overall, participants were satisfied with the 5 video scripts’ content and expressed that they aligned well with their needs and experiences. However, some improvements were suggested that helped to further align the videos with patients’ needs and expectations. For example, participants expressed the need to introduce biologics in a “less scary way,” as “some people are scared from biologics” and to highlight that “biologics are the most effective treatment” for those with IBD. They also found it important to emphasize that patients with IBD should do their own research on the web, discuss their concerns with their provider, and be part of their treatment decision making. In addition, they insisted on reformulating specific parts of the scripts:

you should change from saying healthy diet to essential nutrition;

taking care of yourself shouldn’t be in there. It’s more about you are not always able to control what happens to you;

it's not an isolation that you want, it’s an isolation because you are ill

and changing the representation of a few characters:

I don't think that it’s necessary to show the person dealing with these symptoms as being physically looking a mess.

We then developed 5 high-fidelity video prototypes that incorporated patients’ feedback.

### Phase 3–Implementation Phase

In total, 10 patients with IBD (Table 2) were interviewed to obtain feedback on the high-fidelity video prototypes. We found that participants were generally satisfied with the videos (Multimedia Appendix 4). They reported that they were *informative*, *easy to understand*, *accurate*, and *helpful* and that they were aligned with their perspectives and expectations:

I like the part where they said to write down your symptoms;

being persistent is a good tip;

I like that part of it because your family, friends, and co-workers don’t understand… they don’t see what’s going on, on the inside;

the ending was good, them walking by the bathroom without needing to go in;

it’s very informative. It helps people to put everything in perspective from the medication point of view.

Multimedia Appendix 4 shows specific feedback from patients on how to improve the content and format of each video. For example, participants reported the following:

I don’t think the farting was great, there are many other symptoms;

you should talk to friends and research online. There are communities and group meetings that can be helpful;

adding hobbies that are fulfilling emotionally… painting, music;

emphasizing that it is a difficult disease but that you are not alone;

There are probably non scary ways to show that biologics are injections without like showing needles too.

Their feedback was taken into consideration and integrated into the final products (see Multimedia Appendix 5 for the final videos [19-23]).

## Discussion

We used a human-centered qualitative approach to gain an in-depth understanding of the educational needs of patients with IBD. A series of focus groups and interviews were performed with patients, which informed the iterative development of 5 educational videos optimized for dissemination through social media. First, we explored the patients’ needs and expectations, which informed the development of low-fidelity video prototypes. We then obtained the patients’ feedback and recommendations on these prototypes before developing a set of high-fidelity video prototypes. Finally, we further improved the high-fidelity video prototypes by considering the patients’ specific suggestions for improvements before releasing the final videos. Throughout each of the phases, the data analysis indicated that the 5 videos were aligned with the patients’ needs and expectations. We believe this model can be used to develop other types of patient educational materials, both in IBD and beyond.

In line with previous work, we found that patients have conflicting thoughts about the information on the web and are unsure about the quality of such information [9]. Our data revealed the need to develop and disseminate information from authoritative groups on IBD medical treatment options, the role of diet and nutrition in IBD, how to cope with IBD, how to navigate insurance coverage, and self-advocacy; these are in line with the needs identified previously [7]. Additionally, we identified the need to educate patients’ family, friends, and colleagues, as patients often experience lack of empathy and understanding from their surroundings.

IBD is associated with an increased prevalence of anxiety and depression and feelings of loss of control and social isolation [24,25]. Previous work has shown that medical education and self-management training can decrease disease-related worries and concerns and help patients develop better coping mechanisms [25]. This emphasizes the need to develop educational materials that not only help patients understand their disease but also provide support on how to make informed decisions, develop adaptive coping strategies, and be an effective self-advocate. In addition, good nutrition is important in IBD, as it is estimated that the prevalence of malnutrition is between 16 and 36% in the general IBD population [26,27] and up to 60% in patients with moderate or severe disease [28]. In addition, 76% of the patients with IBD reported that they avoid certain types of food, and 88% believed that nutritional guidance from a health care professional would be beneficial [26]. However, many health care providers report knowledge gaps related to nutrition in IBD [29]. Similarly, although regular exercise is associated with decreased fatigue, improved mental health, and improved quality of life in IBD [30-32], 79% of the patients reported avoiding exercise because of their IBD [29]. Although improving awareness of the importance of nutrition and exercise through the development of educational videos is a first step to address these gaps, more work is warranted to fully understand how to provide patients and providers with the tools they need to eat healthy and stay active.

This study’s main strength is the rigor of the qualitative methodology and the implementation of a design-thinking mindset that guided the development of educational materials. Using a structured design-thinking approach that includes a thorough assessment of human needs, idea generation in brainstorming sessions, prototyping, and testing is fundamental in developing interventions that are feasible, successful, and aligned with users’ needs. Compared with previous studies, our study used a more thorough design-thinking methodology. The rigor of the approach allowed us to build empathy, which was crucial to develop an in-depth understanding of the target population and allowed us to develop educational materials that aligned well with the needs of patients with IBD, as evidenced by the feedback we collected in patient interviews. As for our data collection, 3 team members were present during each of the focus groups, and notes were compared after each session to ensure credibility and reduce the influence of researcher bias on the results. Although qualitative data analysis was performed by only 1 of the researchers, data summaries were presented to all research team members to discuss different perspectives on the insights obtained. Peer debriefing also helped to strengthen the data and improve the quality of the findings. We also obtained iterative feedback from patients throughout the 3 phases, which increased the credibility of our interpretations.

Nevertheless, our study has some limitations. Although our focus group sample was diverse in terms of race, gender, age, diagnosis, and disease duration, focus group participants lacked diversity in terms of education and literacy levels, and many people in our sample used biologics. However, we specifically sought out these missing perspectives in the individual patient interviews, as a key goal of qualitative research is to obtain views from diverse types of patients. Finally, we could not address a key challenge reported by patients: insurance coverage and access to care. In 2018, 8.5% of the US population was uninsured [33], but even with insurance coverage, biologics are frequently denied coverage, and delays are common [34]. Indeed, insurance policies are often not aligned with current IBD guidelines [35]. Although we encourage patients to be proactive about insurance coverage in the educational videos, this is a challenge that can only be addressed by major reforms of the health care system.

### Conclusions

In summary, we used design-thinking methodology to develop 5 IBD-related educational videos for dissemination through social media. This approach led to deep insights and understanding of the unmet educational needs of patients with IBD, which informed the creation of relevant and useful educational materials. This model may be adopted for developing other educational materials in IBD and beyond. Future research will test the impact of educational videos on (1) people’s ability to understand what patients with IBD think and feel and (2) patients’ confidence in self-managing their IBD. The videos will be freely available and will be broadly disseminated on social media using a targeted approach to reach patients with IBD and their family members and friends.

## Appendix

Multimedia Appendix 1 The study design, based on IDEO’s design-thinking model.

Multimedia Appendix 2 Sampling for focus groups and interviews.

Multimedia Appendix 3 Interview guides for phases 1-3.

Multimedia Appendix 4 Patients’ satisfaction and suggestions regarding the content of the video.

Multimedia Appendix 5 The five videos.

## Abbreviations

IBD: inflammatory bowel disease
