# Supplementary material for: Developing Patient-Centered Inflammatory Bowel Disease–Related Educational Videos Optimized for Social Media: Qualitative Research Study
Source: JMIR Med Educ. 2020 Oct 20;6(2):e21639. doi: 10.2196/21639 (PMC7609199; doi:10.2196/21639)
Supplement: Multimedia Appendix 1 [file mededu_v6i2e21639_app1.docx]

**Table 1.** The study design, based on IDEO’s design-thinking model.

| Characteristics | Design-thinking model phases | | | |
| --- | --- | --- | --- | --- |
|  | Phase 1–Inspiration | | Phase 2–Ideation | Phase 3–Implementation |
|  |  | |  |  |
| Aim | Build empathy and insights into IBD^a^ patients’ educational needs | Build empathy and insights into IBD patients’ educational needs | Generate ideas and turn them into video scripts | Iterative updates to the high-fidelity prototypes based on users’ feedback |
| Design | 2 in-person focus groups with 11 patients | 6 phone interviews | 2 in-person focus groups with 12 patients | 10 in-person interviews |
| Sampling | Diverse sample of *typical* patients | Purposive sample of patients with specific circumstances | Diverse sample of patients, including those who participated in phase I | Diverse sample of patients who did not participate in phase I or II |
| Duration | 2 hours | 15-60 mins | 2 hours | 15-30 mins |
| Questions | Open-ended | Open-ended | Close- and open-ended | Close- and open-ended |
| Data collection | Interviews and focus groups were audio recorded and transcribed; research staff also took notes during each session | Interviews and focus groups were audio recorded and transcribed; research staff also took notes during each session | Interviews and focus groups were audio recorded and transcribed; research staff also took notes during each session | Interviews and focus groups were audio recorded and transcribed; research staff also took notes during each session |
| Analysis | Thematic analysis using an inductive approach | Thematic analysis using an inductive approach | Thematic analysis using an inductive approach | Thematic analysis using an inductive approach |

^a^IBD: inflammatory bowel disease.
