# Supplementary material for: Developing Patient-Centered Inflammatory Bowel Disease–Related Educational Videos Optimized for Social Media: Qualitative Research Study
Source: JMIR Med Educ. 2020 Oct 20;6(2):e21639. doi: 10.2196/21639 (PMC7609199; doi:10.2196/21639)
Supplement: Multimedia Appendix 2 [file mededu_v6i2e21639_app2.docx]

**Multimedia Appendix 1 - Sampling for focus groups and interviews**

For the individual phone interviews with 6 ‘extreme’ users we aimed to identify patients with the following characteristics.

1. Someone with a low complicated disease course for over 5 years (no biologic, immunomodulators and in remission).
2. Someone with a high complicated disease course for more than 5 years (failed >1 biologic and required a surgery)
3. Someone newly diagnosed with IBD (<1 year) and needed a quick biologic start
4. Someone with low digital literacy
5. Someone with low general literacy levels
6. Someone on Medicaid

For the focus groups (14 people in total) we recruited patients with the following characteristics to create a diverse group of patients. Assignment to the first or second focus group took place based on availability.

- age groups (18-29, 30-54, or 55 and older): at least 3 in each age group
- male or female: at least 5 of each
- Crohn’s disease or Ulverative colitis: at least 5 with each diagnosis
- Race and ethnicity: At least 2 African Americans, 2 Asians, 2 Hispanics, and at least 2 but no more than 8 Caucasians
