# Supplementary material for: Developing Patient-Centered Inflammatory Bowel Disease–Related Educational Videos Optimized for Social Media: Qualitative Research Study
Source: JMIR Med Educ. 2020 Oct 20;6(2):e21639. doi: 10.2196/21639 (PMC7609199; doi:10.2196/21639)
Supplement: Multimedia Appendix 3 [file mededu_v6i2e21639_app3.docx]

**Multimedia Appendix 2 -- Interview guides**

**Interview Guide - Phase 1**

General questions

1. In a few words, what was the most difficult aspect to deal with since you got diagnosed with IBD?
2. Do you find it difficult to understand what your physician says during a clinic visit?
   1. If yes, what is the most difficult part to understand?
   2. What do you usually do when you don’t understand what the doctor says?
3. What are the best ways to learn about IBD symptoms? IBD causes? And available treatments?
   1. Do you look for information online?
      1. If yes, can you tell us about your experience?
      2. If no, why not?
4. Do you discuss your concerns with your doctor when you need a change in your treatment?
   1. How do you explain these concerns?
   2. If no, why not?

Specific questions related to the use of educational videos

1. Have you already looked up health related educational videos online?
   1. If yes, can you tell us about your experience?
   2. If no, why?
2. What are the best ways to present medical information visually?
3. According to you, what’s the ideal length of time for an online educational video Facebook or other social media sites?
4. What drives you to watch an educational video until the end?
   1. What are you interested in learning?
5. According to you, what are the characteristics of a good educational video?

Specific questions related to self-management and educational videos

1. What would compel you to watch educational videos related to IBD on Facebook or other social media sites?
   1. What are you interested in learning and understanding?
2. How can educational videos make you feel in control of your own health?
3. What about the key content of such videos?
4. How important is it for you to participate in your own treatment decision-making?
   1. How can an educational video help you do that?
5. If thinking about your IBD treatment options makes you anxious, how can education videos help you with this?

**Interview guide – Phase 2**

Questions

1. What are your thoughts on the content of the video?
   1. Is there anything that needs to be added?
   2. Is there anything that needs to be eliminated?
   3. Is the language easy to understand?
2. What are your thoughts on the format?
3. How does the video respond to your needs and expectations? (After listening to the participants’ answers, explore the options below)
   1. Does it improve your knowledge?
      1. In what area does it improve your knowledge?
      2. If no, why not?
   2. Does it reduce your anxiety, fear, or depression?
      1. In what ways does it decrease your anxiety and fear?
   3. Does it improve your self-management skills?
      1. How does it help you self-manage your health?
   4. Does it encourage you to participate in your treatment decisions?
      1. If yes, how so?
      2. If no, why not?
4. How can we improve the videos:
   1. On the content level?
   2. On the format level? (Explore the options below: the visualization of the information? the language used? the colors used? Pictures?)

**Interview guide – Phase 3**

Questions

1. What did you think of the video?
2. Was there anything you did not understand in the video?
3. Was the language easy to understand?
   1. Were there any words in the video that were difficult to understand?
4. What do you think about the music?
5. How can we improve the videos?
   1. Explore changes in phrasing or words
