# Supplementary material for: Developing Patient-Centered Inflammatory Bowel Disease–Related Educational Videos Optimized for Social Media: Qualitative Research Study
Source: JMIR Med Educ. 2020 Oct 20;6(2):e21639. doi: 10.2196/21639 (PMC7609199; doi:10.2196/21639)
Supplement: Multimedia Appendix 4 [file mededu_v6i2e21639_app4.docx]

**Multimedia Appendix 3 - Patients’ satisfaction and suggestions regarding the content of the video**
