# Supplementary material for: Developing Patient-Centered Inflammatory Bowel Disease–Related Educational Videos Optimized for Social Media: Qualitative Research Study
Source: JMIR Med Educ. 2020 Oct 20;6(2):e21639. doi: 10.2196/21639 (PMC7609199; doi:10.2196/21639)
Supplement: Multimedia Appendix 5 [file mededu_v6i2e21639_app5.docx]

**Multimedia Appendix 4 – The five videos**

Video 1
<https://www.youtube.com/watch?v=C8BF4_PZWfI&list=PLRBOHTsQsAZDkNFYd_7Ao4ydzoY0NBVlv&index=2&t=0s>

Video 2
<https://www.youtube.com/watch?v=Xn92mcFQLpU&list=PLRBOHTsQsAZDkNFYd_7Ao4ydzoY0NBVlv&index=2>

Video 3

<https://www.youtube.com/watch?v=KzT1j6ZFpbk&list=PLRBOHTsQsAZDkNFYd_7Ao4ydzoY0NBVlv&index=3>

Video 4

<https://www.youtube.com/watch?v=_OMH4Zk3prw&list=PLRBOHTsQsAZDkNFYd_7Ao4ydzoY0NBVlv&index=4>

Video 5

<https://www.youtube.com/watch?v=uIdSR80eaWw&list=PLRBOHTsQsAZDkNFYd_7Ao4ydzoY0NBVlv&index=5>
